# Modeling joint restoration strategies for interdependent infrastructure systems

**DOI:** 10.1371/journal.pone.0195727

**Published:** 2018-04-12

**Authors:** Chao Zhang, Jingjing Kong, Slobodan P. Simonovic

**Affiliations:** 1 Research Center of Modern Service Science and Technology, Shanghai University of Finance and Economics, Shanghai, China; 2 Shanghai Key Laboratory of Financial Information Technology, Shanghai University of Finance and Economics, Shanghai, China; 3 School of Civil Engineering, Shanghai Normal University, Shanghai, China; 4 Department of Civil and Environmental Engineering, The University of Western Ontario, London, Ontario, Canada; 5 Institute for Catastrophic Loss Reduction, The University of Western Ontario, London, Ontario, Canada; Universidade de Lisboa, PORTUGAL

## Abstract

Life in the modern world depends on multiple critical services provided by infrastructure systems which are interdependent at multiple levels. To effectively respond to infrastructure failures, this paper proposes a model for developing optimal joint restoration strategy for interdependent infrastructure systems following a disruptive event. First, models for (i) describing structure of interdependent infrastructure system and (ii) their interaction process, are presented. Both models are considering the failure types, infrastructure operating rules and interdependencies among systems. Second, an optimization model for determining an optimal joint restoration strategy at infrastructure component level by minimizing the economic loss from the infrastructure failures, is proposed. The utility of the model is illustrated using a case study of electric-water systems. Results show that a small number of failed infrastructure components can trigger high level failures in interdependent systems; the optimal joint restoration strategy varies with failure occurrence time. The proposed models can help decision makers to understand the mechanisms of infrastructure interactions and search for optimal joint restoration strategy, which can significantly enhance safety of infrastructure systems.

## Introduction

The functioning of a society is dependent on large-scale infrastructure systems (e.g. power grid, transportation, water supply network) to deliver services to consumers in an efficient manner. The infrastructure systems are interconnected and interdependent at multiple levels due to their functional needs [1]. The existence of interdependencies can improve operations efficiency of the systems, but can also increase the vulnerability of the systems and the potential for cascading failures [2]. Failure in one infrastructure system can result in service disruptions in other systems [3]. One typical example is the 2003 power blackout in the US and Canada. The large scale power outage caused traffic congestion and the disruption of water supply and communications. Given the increasing impact of natural and man-made disasters on infrastructure systems, it is critical to restore the systems jointly to minimize the impact of the disasters.

Restoration activities are essential for infrastructure systems to recover from a disruption [4]. Failed infrastructure components should be prioritized and restored to avoid additional instability. Problems related to restoration of infrastructure systems have been widely studied. Most of the work done concentrated on restoration strategies for single infrastructure system. From the network analysis perspective, Liu et al. [5] presented a modeling framework to study the effect of different restoration strategies on robustness of a system against cascading failures. To minimize potential hurricane damages, Arab et al. [6] proposed a stochastic integer programming model for restoration resource allocation to electric power system. With the objective of enhancing resilience of transportation network, Vugrin et al. [7] formulated a two-level optimization model to identify the optimal recovery modes and sequences after disruptions. Using colored Petri nets, Luna et al. [8] developed a model to improve post-earthquake restoration process of a water distribution system. Subject to limited available repair resources, Wang et al. [9] formulated an optimization model to determine the optimal recovery process for internet protocol network after disruptions. Above work represents restoration studies for different infrastructure types, as well as a variety of approaches.

As current infrastructure systems often exhibit multiple interdependencies, there are studies of restoration strategies for interdependent infrastructure systems. Some of them provide restoration suggestions at system level, such as how to allocate restoration resources to different infrastructure types. MacKenzie et al. [10] proposed static and dynamic decision models to determine the optimal resource allocation to facilitate the recovery of impacted infrastructure industries by minimizing production losses. Zhang et al. [11] presented an approach for allocating restoration resources to enhance resilience of interdependent infrastructure systems, in which the recovery process of interdependent infrastructure systems is described as the dynamic inoperability input-output model.

Other studies focused on how to determine the restoration sequence of disrupted infrastructure components. To minimize the cost of network flow after a disaster, Lee et al. [12] developed a mixed-integer, network-flow based model to identify which components should be restored or reconstructed. Using mixed integer linear programming, Cavdaroglu et al. [13] developed a mathematical formulation that integrates the restoration planning and scheduling decisions to restore essential services provided by interdependent infrastructure systems. To maximize the cumulative weighted flow in infrastructure networks, Nurre et al. [14] proposed an integrated network design and scheduling problem which models real-time restoration activities and long-term scenario planning activities. Based on the proposed interdependent network design problem, Gonz´alez et al. [15] developed a model to optimize the resource allocation and recovery strategy in interdependent infrastructure networks with consideration of the savings due to simultaneous collocated recovery. However, in above studies, there were no obvious differences among the operating models of different infrastructure. For example, the difference in the service transmission speeds of different systems has not been taken into consideration. Coffrin et al. [16] studied the restoration problem by solving a mix integer linear programming model to maximize the sum of infrastructure service demands, while they did not take component repair time into consideration. To enhance resilience of infrastructure systems, Ouyang and Wang [17] studied the joint restoration with five types of the restoration strategies proposed in advance.

Since the failure propagation and recovery processes of infrastructure systems are affected by their interdependencies [18], the interconnected relationships among infrastructure systems should be modeled first when developing restoration strategies. Network based methods [19, 20] have been extensively applied to address this problem. Related work includes topology based methods and network flow based methods [21]. The former model the interaction process among infrastructure systems mainly considering their physical connection structure [22–24]. The latter consider the services made and delivered by infrastructure systems together with their structure [25–29]. Flow based methods can capture service flow characteristics of infrastructure systems, and provide more realistic descriptions of their operation mechanisms. However, the flow based models require more detailed infrastructure data, such as parameters of infrastructure operation models, which are usually not easy to obtain.

The objectives of existing restoration studies based on network methods include minimizing the number of failed nodes, minimizing the connectivity loss of network [5], minimizing the fraction of consumers affected [8], maximizing system resilience [7,17], maximizing network flows [14], and/or minimizing various costs and economic losses [12,15]. In practice, decision makers typically favor use of the total economic loss to evaluate the impacts of a disaster. The occurrence time of infrastructure failures impacts the economic loss directly, e.g. the power outage happening in the morning or evening could cause different social and economic impacts. So, the occurrence time is an essential factor for effective restoration strategy development, while it is seldom considered in existing studies.

The objective of this paper is to present an approach that enables development of effective joint restoration strategy for interdependent infrastructure systems after a disruptive event, i.e. determining the joint restoration sequence of disrupted infrastructure components. The contributions of the study include:

1. Assessment of the total economic loss of interdependent infrastructure system considering the occurrence time of individual infrastructure failures. Minimum of the total economic loss is used as the objective of system restoration.
2. Development of a model for describing interaction processes among interdependent systems considering failure types, infrastructure operating rules and interdependencies among systems. The presented model can capture the change of service of each infrastructure system during restoration process, which is used to examine the effect of restoration activities at each time step.
3. Use of the model to propose an effective restoration strategy by integrating the restoration objective and interdependent infrastructure system interaction processes and consideration of the restoration time of disrupted infrastructure components.

The rest of the paper is organized as follows: Section 2 presents the models for describing the structure of interdependent systems and their interaction process. Section 3 presents a model for the development of restoration strategy at infrastructure component level and a numerical method for solving the model. In Section 4, the proposed models are evaluated systematically using an electric-water system example by simulation. The sensitivities of the results to model parameters are also analyzed. Section 5 draws the conclusions and gives the direction for future research.

## Modeling of interdependent infrastructure systems

This section presents models for describing (i) interdependent infrastructure system structure and (ii) interaction process, which are the key building blocks for the development of restoration strategy.

### Representation of interdependent infrastructure systems

This research applies a network-based approach to describe the topology of interdependent infrastructure systems [30]. In the network, nodes correspond to facility components of an infrastructure system, and links represent the physical elements connecting the nodes. Electric power and water supply systems are selected for illustrative purposes. For electric power network, power plants and substations are represented as nodes, transmission lines between nodes are described as links [31]. For water supply network, water plants and pumping stations are represented as nodes, and the pipes between the nodes are described as links. Infrastructure network can be enriched by assigning different roles to different nodes. Based on functional features, nodes in infrastructure networks can be classified as source nodes and load nodes. Services are delivered from source nodes to load nodes through links, and then transmitted to the consumers. The directions of links can denote the transmission directions of services. In electric power and water supply networks, power plant and water plant nodes are source nodes; substation nodes and pumping nodes are load nodes.

**Fig 1:**
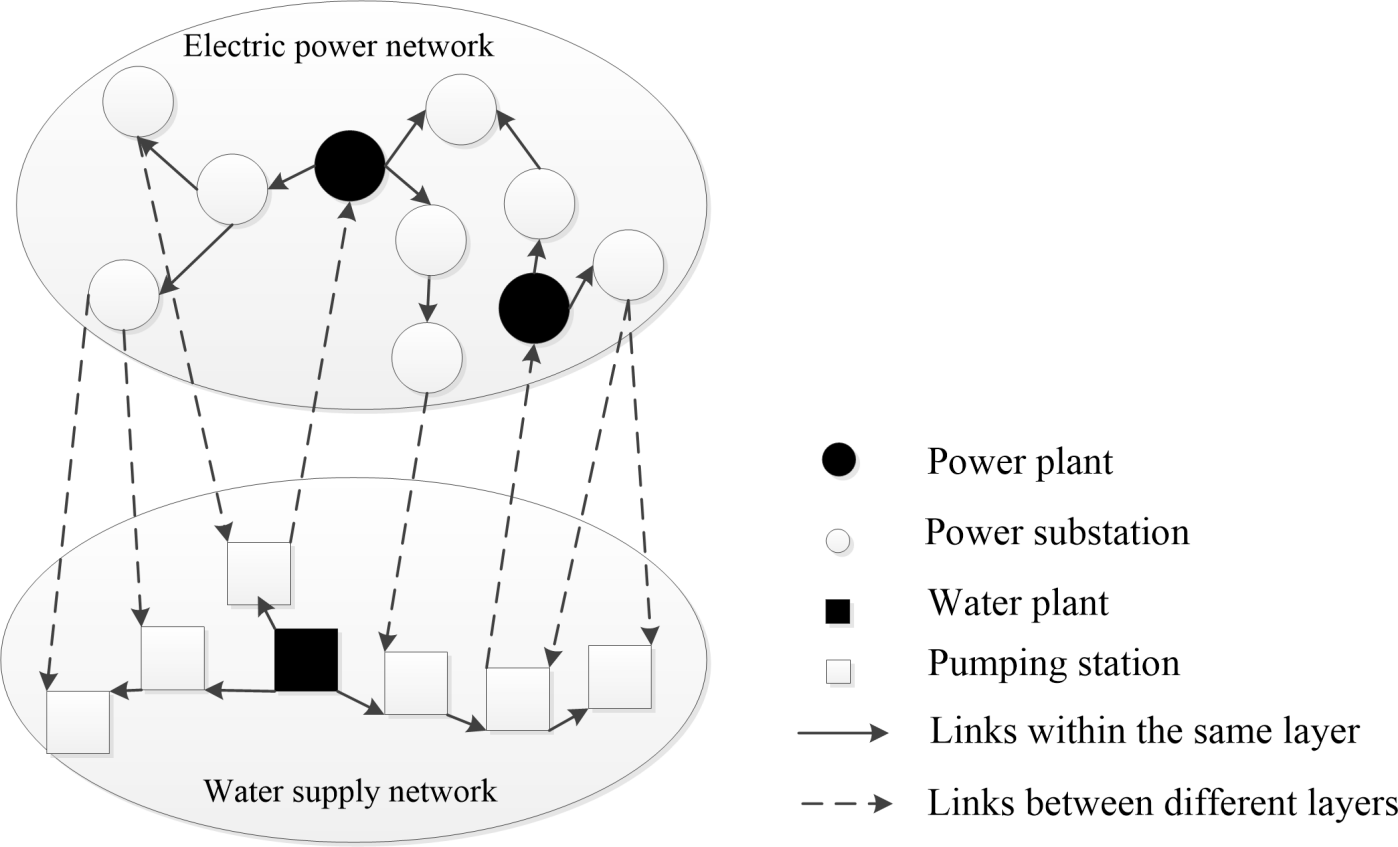
Diagram of interdependent infrastructure networks.

The interdependencies between electric power and water supply networks are assumed as [32]: (i) power plant nodes require water input from pumping nodes for cooling; and (ii) water plant nodes and pumping nodes need power input from substation nodes to maintain their operations. Multilayer network is used to describe interdependent infrastructure systems [33]. Fig 1 illustrates an interdependent system. Each subsystem is described as a network layer, nodes and links in the same layer belong to the same infrastructure subsystem; links crossing layers denote service dependencies between nodes in different subsystems.

### Operating models of infrastructure systems

Operating rules of infrastructure systems should be considered in the study of interaction process after a disruptive event. Several models have been developed to describe the operation of electric power and water supply systems. For electric power system, the ORNL-PSerc-Alaska (OPA) model [34] and Crucitti-Latora-Marchiori (CLM) model [35] are widely used. The former offers a realistic representation of a power system, which results in notable computational limitations, requires a more involved process in setting parameters, and has multiple parameters. CLM is designed to study common features of flows in many systems. It offers advantages for modeling cascading failures with fewer parameters, while is unable to directly represent generator capacity and load demand. Simulation model [36] and dynamic flow model [37] have been used to describe the operating rules of water supply system.

Due to lack of detailed data of infrastructure systems, this study only considers the essential functional properties of the selected systems, and applies a network flow model to describe the operating rules. In the model, only node failures are considered. According to the cause of failures, the node failures are divided into two types. The first one is physical failure, which means the nodes failed due to direct physical damages. They cannot recover without restoration activities. The second type is functional failure, which means the nodes failed due to indirect impact or insufficient service input. For each node, there is a functional threshold denoting the minimum level of different service inputs required for its operation. If one type of service input is below the threshold, the node will functionally fail; if the service input is regained and above the threshold level, the node functionality will be recovered.

The water supply network is composed of water plant nodes, pumping nodes and links between the nodes. The network flow model for water supply network includes the following rules: (i) At initial time step, the water input of a pumping node is equally supplied by the connected water plant nodes (there is at least one path between the pumping node and the water plant node). If a pumping node is connected with *n* plant nodes, each plant node is responsible for *1/n* water input. (ii) The water delivery between a plant node and a pumping node is through the shortest path between them and the water delivery through each link in the path needs one time step. (iii) Nodes are not functional if they encounter physical failures or functional failures. (iv) Links connected to the failed node are not functional. (v) The water input of the pumping node will decrease, as all paths between the pumping node and a plant node are not functional. (vi) The pumping node is not functional (failure) if its water input is below the functional threshold level. (vii) Functionally failed nodes can recover if their services are re-established and the inputs are above the functional thresholds.

The electric power network is composed of power plant nodes, substation nodes and links. The network flow model for electric power network is similar to that for water supply network but different in one point: as power transmission is fast, the delivery of power between each pair of nodes is instantaneous.

The rules for interdependencies between electric power and water supply networks are as follows: (i) The water input of a power plant node is supplied by the nearest pumping node. If a power plant node does not receive water input, it will functionally fail. (ii) The power input of a water node is provided by the nearest substation node. Considering service buffers, each water node has a backup power, which can maintain its operation for one time step when power input is insufficient. If a water node cannot obtain the power input and its backup power has been used, the water node will functionally fail.

### Interaction process of interdependent infrastructure systems

**Fig 2:**
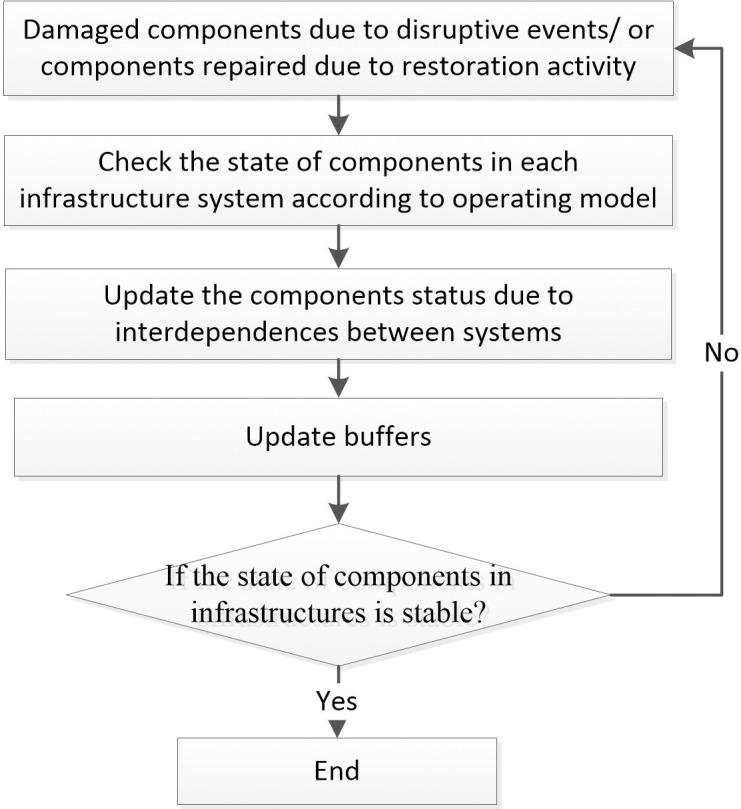
Analysis flowchart for interaction process.

If some nodes in infrastructure networks physically fail, due to service dependencies, the failures could propagate within and across interdependent infrastructure networks. Considering that the restoration activities would be implemented, the state change of infrastructure nodes can be captured by applying an iterative procedure. An analysis flowchart for describing the interaction process at each time step is shown in Fig 2.

For electric power and water supply networks, procedures in Fig 2 are described as follows:

1. Update the set of physically failed nodes in two networks. For the initial time step, identify the physically failed nodes and remove them from the networks; for other time steps, if some physically failed nodes are restored, add them back to the networks.
2. Check the state of nodes in each infrastructure network separately. Based on the set of physically failed nodes, and the set of functionally failed nodes, the operating model is applied to update the state of each node in networks. For electric power network, the power delivery is instantaneous. Verify whether there are some broken paths between plant nodes and substation nodes, or some interrupted paths are reconnected; if yes, compute the power input of each node. If the power inputs of some substation nodes are below the functional threshold level, modify the state of these nodes into functional failure; if some functionally failed nodes regain enough power input, change the state of these nodes to normal. The analysis process for water supply network is similar. One difference is that the water delivery through each link needs one time step, so the state change of the water nodes is relatively slow.
3. Update states of nodes according to their interdependencies. Based on the state of nodes in step (2), according to the interdependencies between networks, for each node, compute the service input from the other network. Identify the functioning nodes which cannot obtain sufficient service input, or the functionally failed nodes which regained enough service input from the other network. In particular, as each water node has a backup power, if a water node cannot obtain sufficient power input for the first time, the node will not functionally fail immediately. It may fail at the next time step.
4. Update buffers. Any existing buffers, such as the remaining capacity of power backup for water nodes are updated, if necessary. If there are any changes in nodes’ states at this time step, or more restoration is planned to be adopted at the next time step, a new iteration is then initiated by starting from step (1) again; else if all nodes in two networks are functional, end.

**Fig 3:**
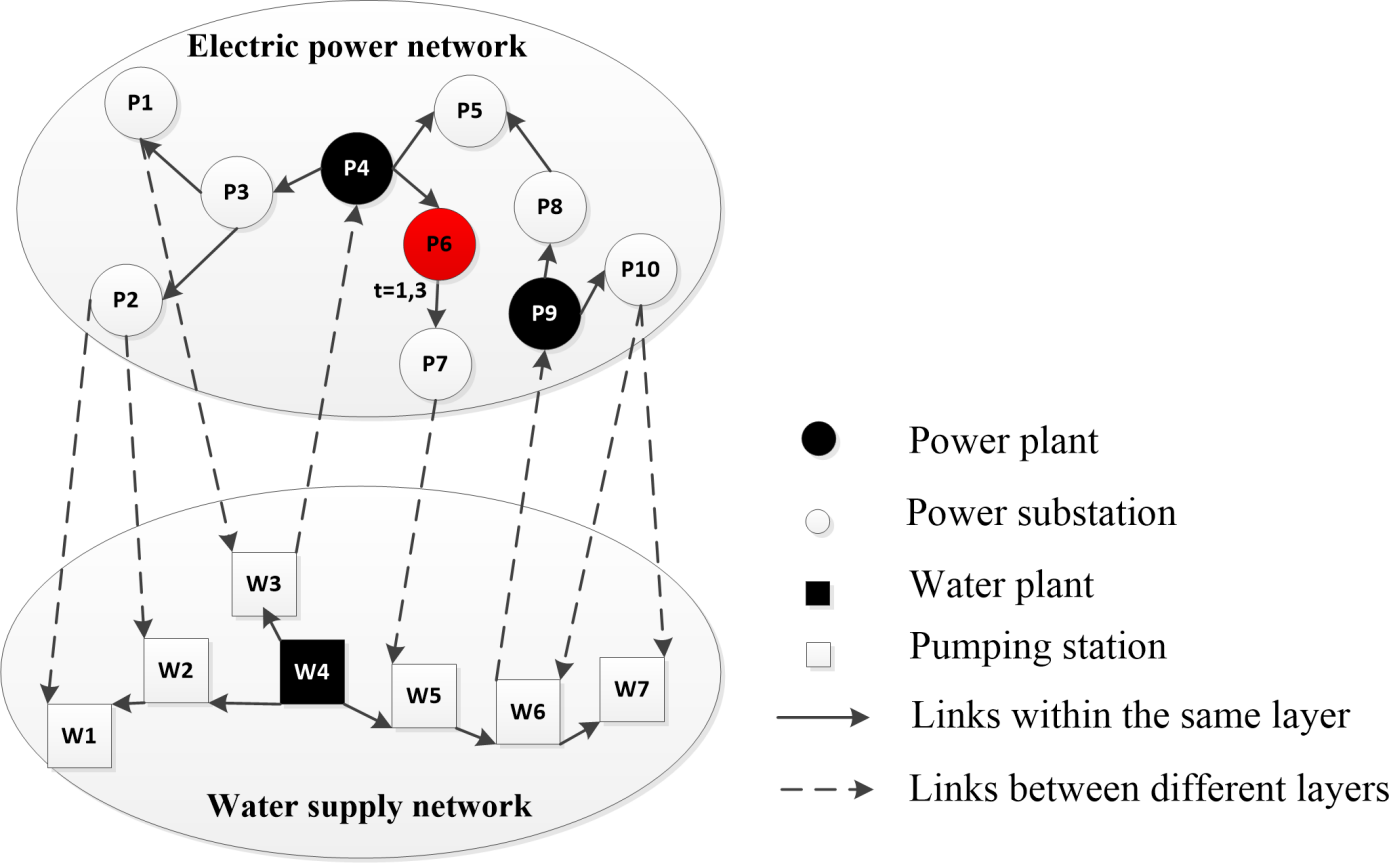
An illustrative example of the interaction process. Substation node P6 physically fails at t = 1, and it is restored at t = 3.

Given the interaction process model described above, the consequences of physical failures and restoration activities can be evaluated. For example, in Fig 3, let us assume that substation node P6 physically fails at t = 1 and is restored at t = 3, the consequences of the interaction process at each time step are shown in Table 1.

**Table 1:** Consequences of the interaction process.

Time step t	Set of physically failed nodes	Set of functionally failed nodes	Change in capacity of power backup for water nodes
1	{P6}	{P7}	W5: 1→0
2	{P6}	{P7, W5}	
3	{}	{W6}	W5: 0→1
4	{}	{P9, W7, P8, P10}	
5	{}	{}	

In Table 1, (i) at t = 1, substation node P6 physically fails. Since power transmission is instantaneous, substation node P7 functionally fails due to lack of power input. The power backup is used at this time step for pumping station node W5. (ii) at t = 2, without restoration activity, P6 is still physically failed. Due to lack of power, W5 is added into the set of functionally failed nodes. (iii) at t = 3, substation node P6 is restored and the set of physically failed nodes becomes empty. Functionally failed nodes P7 and W5 recover to normal operations since they regain the necessary power input. The power backup for W5 is reinstated again. As the water delivery requires one time step, the pumping node W6 becomes functionally failed due to lack of water input. (iv) at t = 4, W6 recovers to normal operation since it regains the water input. However, as water delivery requires one time step, power plant node P9 and pumping station node W7 functionally fail due to lack of water input. Substation nodes P8 and P10 functionally fail because of the failure of P9. (v) at t = 5, all functionally failed nodes are recovered and the set of functionally failed nodes becomes empty.

## Joint restoration strategy model

After a disruptive event, restoration activities would be conducted to recover physically failed infrastructure nodes. To minimize the economic loss from the infrastructure failures, this section presents a model to develop joint restoration strategies.

### Metric for impact of infrastructure failures

Decision makers typically favor the economic loss in evaluating the impact of a disaster. This paper uses the economic loss from the infrastructure failures as the metric of their impact. The economic loss consists of the consumer loss due to lack of infrastructure service, and the restoration cost of physically failed nodes. For electric power network, we assume nodes have equal service capacity, that is, the number of consumers served by each node is the same. When some power nodes physically fail at *t*_0_, *L*_*p*_(*t*) represents the economic loss of the network at time *t*>*t*_0_, and is expressed by Eq (1).

$$L_{p}(t)=C_{p}(t)+R_{p}(t)$$ (1)

where *C*_*p*_(*t*) represents the consumer loss due to lack of power service, *R*_*p*_(*t*) denotes the restoration cost.

**Fig 4:**
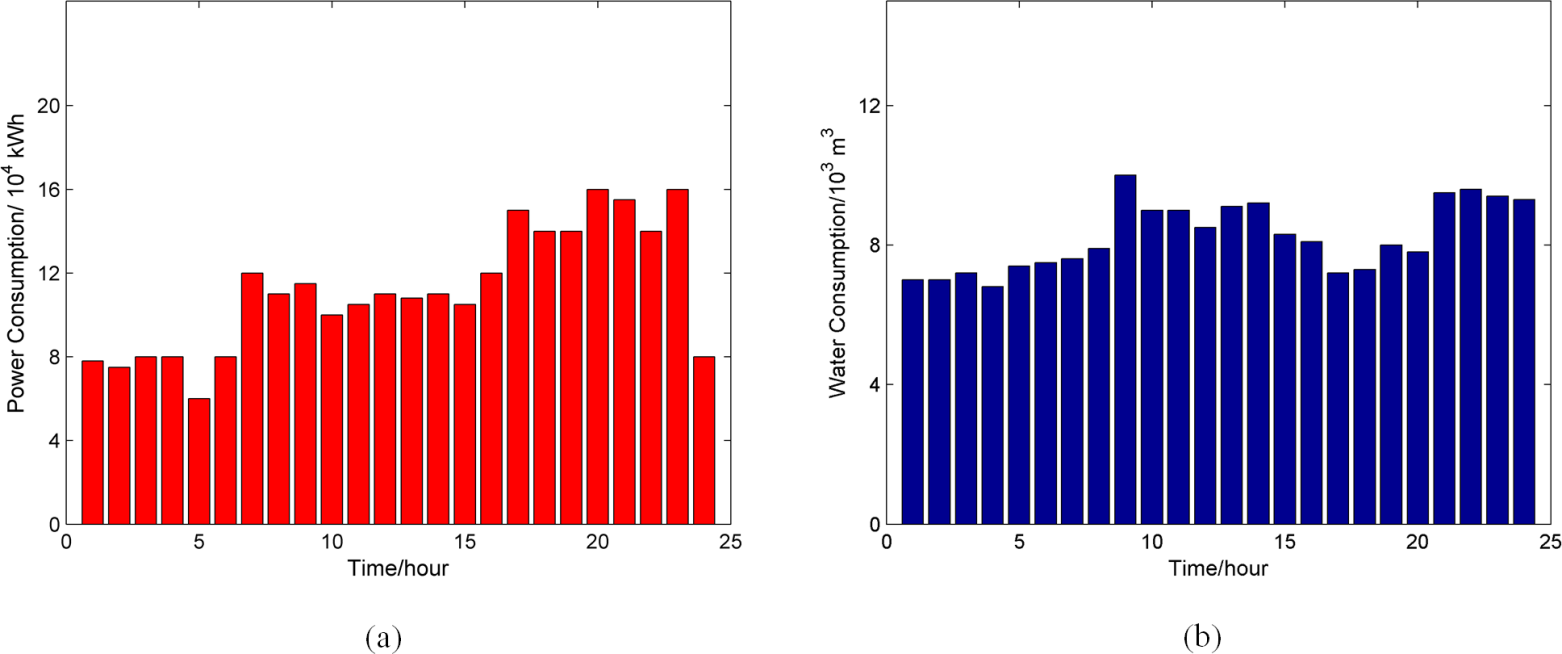
Average hourly service consumption of Guilin in China. (a) electric power; (b) water.

Fig 4 shows the average hourly power and water consumption of the Guilin City in China (data derived from government survey). It can be seen that obvious difference exists in power consumption at different time of the day. The highest is about 160,000 kWh, while the lowest is only about 50,000 kWh. Therefore, for the same failures, the consumer loss is dependent on the failure occurrence time. Since the service capacity of each power node is the same, the consumer loss at time step *t* can be expressed by Eq (2).

$$C_{p}(n_{p}^{t},t)=\varphi_{p}\times c_{p}^{t}\times (n_{p}^{t}/N_{p})$$ (2)

where *φ*_*p*_ represents the hourly consumer loss for a unit power loss; $c_{p}^{t}$ is the power consumption at *t*; $n_{p}^{t}$ is the number of failed power nodes at *t*; *N*_*p*_denotes the number of nodes in the network.

In Eq (1), *R*_*p*_(*t*) denotes the restoration cost. In a real world context, it is very hard to repair the infrastructures immediately after the disaster due to the extent of the damage and availability of resources for recovery. With the increasing recovery of damaged infrastructures, it is easier to repair the rest of damaged infrastructure as the restoration resources are easier to obtain. So, the average of early restoration cost for the same damaged infrastructure is always higher than the later restoration cost. The assumption introduced here is that the restoration cost is negatively correlated to the time difference between failure occurrence and restoration activities, and is expressed as

$$R_{p}(k_{p}^{t},t)=\theta_{p}\times k_{p}^{t}/(t-t_{0})$$ (3)

where *θ*_*p*_ represents the unit cost of restoring a physically failed power node; $k_{p}^{t}$ denotes the number of restored physically failed nodes at time step *t*.

$$L_{w}(t)=C_{w}(t)+R_{w}(t)$$ (4)

Similarly, the expression of the economic loss for water supply network at time step *t* is shown in Eq (4), where *C*_*w*_(*t*) is the consumer loss due to lack of water supply, *R*_*w*_(*t*) is the restoration cost.

Therefore, the economic loss of electric-water network at *t* is given in

$$L(t)=L_{P}(t)+L_{W}(t)$$ (5)

## Model of restoration process

The objective of restoration activities is to minimize the economic loss from the infrastructure failures over time. To develop joint restoration strategy at component level, i.e. determining the joint restoration sequence of physically failed nodes at each time step, following assumptions are made: (i) the earliest beginning of restoration is the time step following infrastructure failures occur. (ii) A physically failed nodes could recover to normal functioning within one time step when they are restored. (iii) For each infrastructure network, the time steps used for restoration should be no more than the number of physically failed nodes in the network.

For electric-water networks, if physical failures occur at *t*_0_, we use *F*_*p*_ and *F*_*w*_ denote the sets of physically failed nodes in two networks at *t*_0_. $s_{p}^{t}\subset F_{p}^{}$ and $s_{w}^{t}\subset F_{w}^{}$ represent the set of restored nodes at time step *t*. The restoration strategy can be written as

$$S=\{\{s_{p}^{t_{0}+1},s_{w}^{t_{0}+1}\},\{s_{p}^{t_{0}+2},s_{w}^{t_{0}+2}\},\ldots \}$$ (6)

where $\left\{s_{p}^{t_{0}+i},s_{w}^{t_{0}+i}\right\}$ is the pair of sets of restored nodes in two networks at time step *t*_0_+*i*,where *i* = 1,2,…. As water delivery is slow, when all physically failed nodes are restored, additional time is required for functionally failed nodes to recover back to normal. Let *d* denote a large enough number, all nodes will recover to normal functioning within time *t*_0_+*d*. According to the representation of economic loss in each time step (see Eq (5)), the optimal joint restoration strategy is the solution to following optimization problem:

$$\min\;z=\sum_{t=t_{0}}^{t_{0}+d}\left(L_{p}(t,k_{p}^{t},n_{p}^{t})+L_{w}(t,k_{w}^{t},n_{w}^{t})\right)$$ (7)

$$s.t.\;s_{p}^{t_{0}}\cup s_{p}^{t_{0}+1}\cup \ldots \cup s_{p}^{t_{0}+d}=F_{p},\;s_{w}^{t_{0}}\cup s_{w}^{t_{0}+1}\cup \ldots \cup s_{w}^{t_{0}+d}=F_{w}$$ (8)

$$s_{p}^{t_{0}}\cap s_{p}^{t_{0}+1}\cap \ldots \cap s_{p}^{t_{0}+d}=\emptyset ,\;s_{w}^{t_{0}}\cap s_{w}^{t_{0}+1}\cap \ldots \cap s_{w}^{t_{0}+d}=\emptyset$$ (9)

$$s_{p}^{t_{0}+l}=\emptyset ,if\;|F_{p}|<l\leq d$$ (10)

$$s_{w}^{t_{0}+g}=\emptyset ,if\;|F_{w}|<g\leq d$$ (11)

where $k_{p}^{t}{=|s}_{p}^{t}|$, $k_{w}^{t}{=|s}_{w}^{t}|$, denotes the number of restored physically failed nodes in two infrastructure networks at time step *t*; vector $n_{p}^{}=(n_{p}^{t_{0}},n_{p}^{t_{0}+1},\ldots ,n_{p}^{t_{0}+d})$ and $n_{w}^{}=(n_{w}^{t_{0}},n_{w}^{t_{0}+1},\ldots ,n_{w}^{t_{0}+d})$ denote the number of failed nodes in two networks at each time step after failures occur, elements in the vector could be acquired by applying proposed interaction process model with initial conditions *F*_*p*_,*F*_*w*_, and restoration strategy *S*. The objective function (7) is to minimize the total economic loss due to infrastructure failures. Eq (8) ensures all the physically failed nodes will be restored. Eq (9) ensures each node is a member of only one set of restored nodes. Eq (10) and Eq (11) ensure that the number of time steps used for restoration is not greater than the number of physically failed nodes, assumption (iii).

### Solution method for the programming model

The joint restoration strategy model (Eqs (7)–(11)) is a multistage decision problem, and the solution is the optimal joint restoration sequence of the physically failed nodes. As the interaction process among interdependent infrastructure networks is nonlinear and complex, the model is hard to solve with standard optimization techniques. This section proposes a numerical method for solving the model by applying genetic algorithm (GA) [38]. The search procedure for an optimal restoration sequence is performed according to the following steps.

1. Code design. Enter the number of physically failed nodes and express a restoration sequence by a genotype, which is a 0–1 variable matrix $G={\left[g_{ij}\right]}_{K\times (K_{1}+K_{2})}$, in which *K*_1_ = |*F*_*p*_| (the number of physically failed nodes in electric power network), *K*_2_ = |*F*_*w*_| (the number of physically failed nodes in water supply network), and *K* = max(*K*_1_,*K*_2_). Matrix *G* is subject to the following constraints:
  $$\left\{ \begin{array}{l} \sum_{i=1}^{K}\sum_{j=1}^{K_{1}}g_{ij}=K_{1}\\ \sum_{i=1}^{K}\sum_{j=K_{1}+1}^{K_{1}+K_{2}}g_{ij}=K_{2}\\ \sum_{i=1}^{K_{}}g_{ij}\leq 1,\;for\;j=1,2,\ldots ,K_{1}+K_{2} \end{array}\right.$$ (12)
  where: for *j*≤*K*_1_,
  $$g_{ij}=\left\{ \begin{array}{l} 1,\;\mathrm{the}j‑th\mathrm{physically}\;\mathrm{failed}\;\mathrm{node}\;\mathrm{in}\;\mathrm{electric}\;\mathrm{power}\;\mathrm{network}\;\mathrm{is}\;\mathrm{restored}\;\mathrm{at}\;\mathrm{time}\;t_{0}+i\\ 0,\;\mathrm{the}\;j‑th\mathrm{physically}\;\mathrm{failed}\;\mathrm{node}\;\mathrm{in}\;\mathrm{electric}\;\mathrm{power}\;\mathrm{network}\;\mathrm{is}\;\mathrm{not}\;\mathrm{restored}\;\mathrm{at}\;\mathrm{time}\;t_{0}+i \end{array}\right.$$
  for *K*_1_<*j*≤*K*_1_+*K*_2_,
  $$g_{ij}=\left\{ \begin{array}{l} 1,\;\mathrm{the}\;(j-K_{1})‑th\mathrm{physically}\;\mathrm{failed}\;\mathrm{node}\;\mathrm{in}\;\mathrm{water}\;\mathrm{supply}\;\mathrm{network}\;\mathrm{is}\;\mathrm{restored}\;\mathrm{at}\;\mathrm{time}\;t_{0}+i\\ 0,\;\mathrm{the}\;(j-K_{1})‑th\mathrm{physically}\;\mathrm{failed}\;\mathrm{node}\;\mathrm{in}\;\mathrm{water}\;\mathrm{supply}\;\mathrm{network}\;\mathrm{is}\;\mathrm{not}\;\mathrm{restored}\;\mathrm{at}\;\mathrm{time}\;t_{0}+i \end{array}\right.$$
  Comprehensively considering convergence speed and the variety of individuals, some genotypes of initial individuals are chosen from feasible solutions, the others are randomly generated.
2. Compute the fitness value. The fitness value (objective function) is the total economic loss over time. For each genotype, which corresponds to a restoration sequence, the fitness value of Eq (7) can be calculated by applying the developed interaction process model with initial conditions. For genotypes that do not meet constraint (12), we use a large enough number as a penalty of unacceptable solution.
3. Rules for selection, crossover, mutation and stopping. The roulette method, two-point crossover and random mutation are chosen as rules for the selection, crossover and mutation. There are two rules for stopping, one is the number of maximum generations, and the other is the convergence of the optimal fitness value between generations. When algorithm stops, the genotype corresponding to the optimal fitness value is the optimal restoration strategy.

## Case study

The models developed in this paper are tested using an illustrative case study of electric-water system.

### Generation of infrastructure system topology

The topology generator introduced by Ouyang et al. [39] is applied to construct infrastructure network topology. The network layers are created based on the spatial proximity of nodes during the network growth. Specifically, for electric power network, following procedure is used to generate the network: (i) the network is seeded with several independent nodes as plant nodes and no links among them. (ii) At each time step, a new substation node is added to the network, at least one new link is connected to the node, the other end of the link is connected to existing node in the network based on the minimum Euclidean distance. In addition, there is a probability *γ* of adding another new link connecting the new node with the second nearest existing node. (iii) After the final time step, a sparse random network is generated.

**Fig 5:**
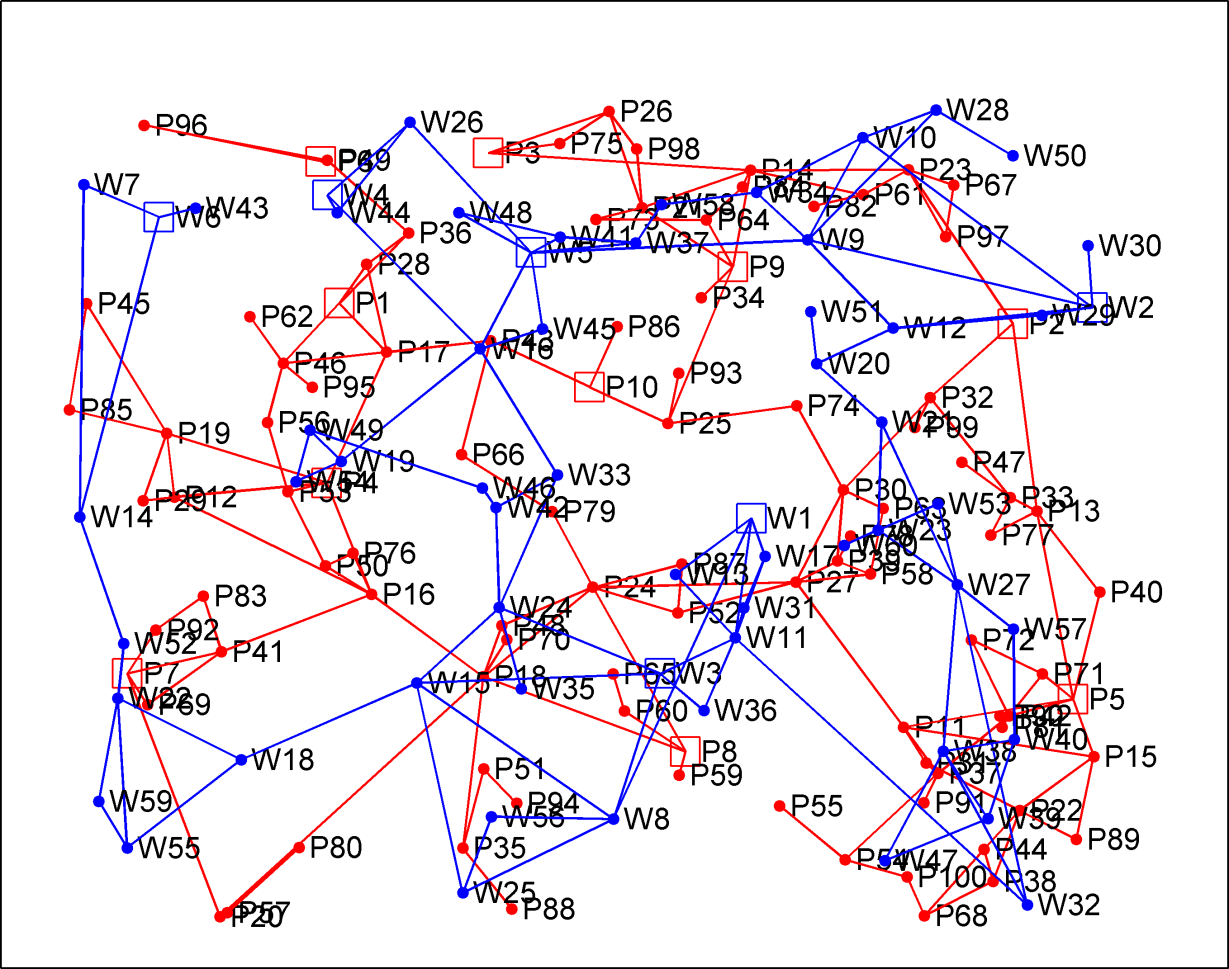
The electric power network and the water supply network. The red squares nodes (P1-P10) are the power plant nodes, while the blue squares nodes (W1-W6) are the water plant nodes. The red lines are electric transmission lines, and the blue lines are water pipelines.

Referring to average degree [40] of real infrastructure networks, i.e. American grid (2.78) [41] and grid in northeast China (2.36) [42], the probability parameter *γ* is set as 0.5, and aim to generate infrastructure networks with average degree close to 2.5. Based on the presented procedure, the electric power and water supply networks are generated in the same graph (see Fig 5). Specifically, the electric power network includes 100 nodes and 148 links, 10 of the nodes are plant nodes (blue squares, P1–P10), the others (P11–P100) are substation nodes. The water supply network includes 60 nodes, 6 of them are plant nodes (red squares, W1–W6), the others are pumping station nodes (W7-W60).

### Failure propagation analysis

The interaction process among interdependent infrastructure systems is first investigated. The service dependencies between power and water nodes are as follows: (i) the water input of a pumping node is equally provided by the connected water plant nodes; (ii) the power input of a water node is supported by the nearest power node. For simplification, functional thresholds for different types of nodes are set to 0.5.

**Fig 6:**
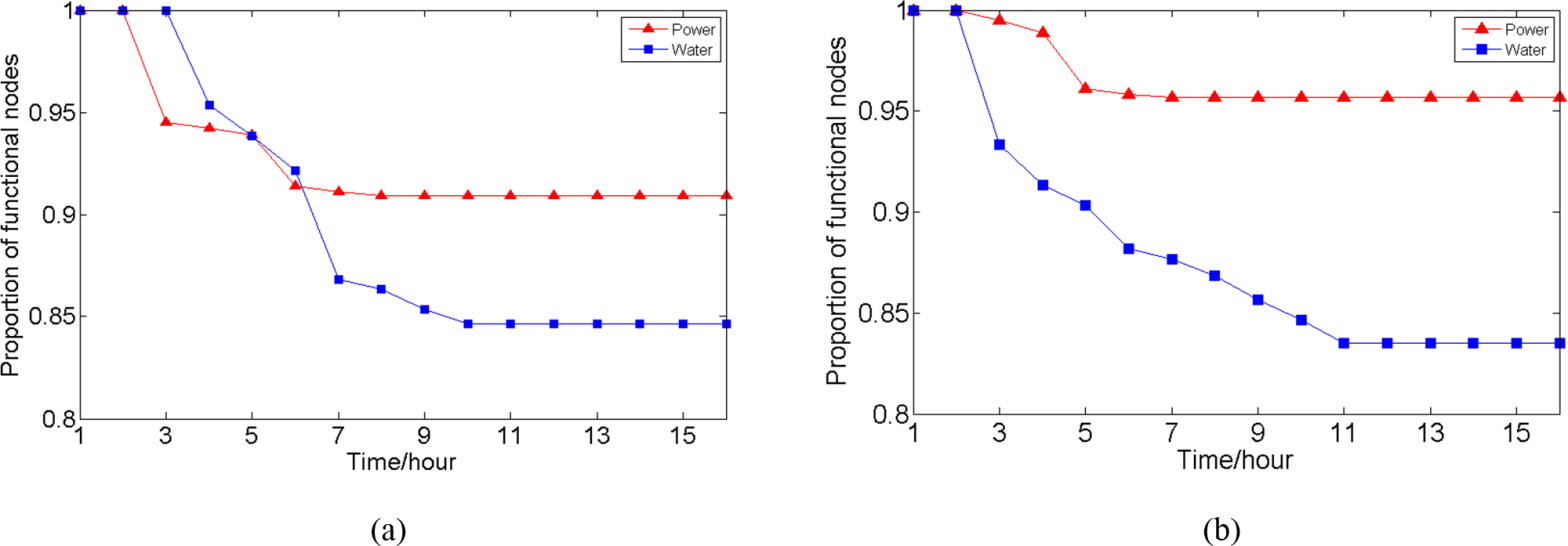
Numbers of functional nodes in two networks at each time step. (a) Initial node failures occur in the power network; (b) initial node failures occur in the water network.

For the analysis, we set 1 time step to be 1 hour. To investigate interaction process, we use the proportion of functional nodes in the network as infrastructure performance measure. Suppose some power nodes physically fail, the simulation process is as follows: At time t = 2, randomly choose four power nodes to physically fail and remove them from the network. Without restoration activities, according to the interaction process model, the proportion of functioning nodes in two networks at each time step is calculated and shown in Fig 6(A). The result is averaged over 50 runs for random physical failures. The simulation process for water nodes is similar, and the result is shown in Fig 6(B).

In Fig 6(A), as the power transmission is instantaneous, the physical failure of nodes result in functional failure of some other power nodes, an immediate decrease is seen in the proportion of functioning power nodes at t = 2. Since there are power backups for water nodes, the proportion of functioning water nodes begins to decrease at t = 3. As time passes by, if any water nodes cannot provide sufficient water to power plant nodes, the corresponding power plant nodes will functionally fail, which further causes decrease in the proportion of functional power nodes, and a sharp decrease is seen at t = 6. At t = 7, there is another obvious decrease in the proportion of functional water nodes. The time delay for the state change in water nodes is due to the existence of power backup. Finally, the proportion of functional power nodes becomes stable at t = 8, and decreases to about 0.91. The failure propagation in water network is relatively slow. The proportion of functional water nodes becomes stable at t = 10, and decreases to about 0.85. If water nodes physical failures occur (see Fig 6(B)), the proportion of functional power nodes begins to decrease at t = 2, and has a sharp decrease at t = 5. The cause is due to the failure propagation in water network, the water inputs of some power plant nodes become insufficient. These nodes functionally fail, and induce the decrease in proportion of functional power nodes. Finally, the decrease in the proportion of functional nodes for water is about 0.16, while for power is less than 0.05.

The results show that physical failures in one infrastructure network can cause more failures in interdependent infrastructure networks. Besides, though there are power backups, the interdependencies still have much larger effect on water network than on the power network. The simple explanation is that more water nodes depend on power service. Although the results rely on the interdependent structure and model parameters, the analysis can help managers achieve a better understanding of the interaction mechanism of interdependent infrastructure systems.

### Joint restoration strategy analysis

To obtain the optimal joint restoration strategy for infrastructure failures, the parameters defined in the joint restoration strategy model should be provided first. In this section, it is assumed that the hourly power and water consumption in each hour during a day is equal to the data shown in Fig 4, and calculated with assumed units, e.g. the power and water consumption at time step t = 1 is 7.82 power units and 7.12 water units. The given parameters in joint restoration strategy model are shown in Table 2. As electric power system is more essential to our society, parameters for power network are set to be double of that for water network.

**Table 2:** Parameters in joint restoration strategy model.

	Electric power	Water supply
**Unit cost of restoring a physically failed node**	*θ*_*p*_ = 2	*θ*_*w*_ = 1
**Hourly consumer loss for a unit service loss**	*φ*_*p*_ = 20	*φ*_*w*_ = 10

**Fig 7:**
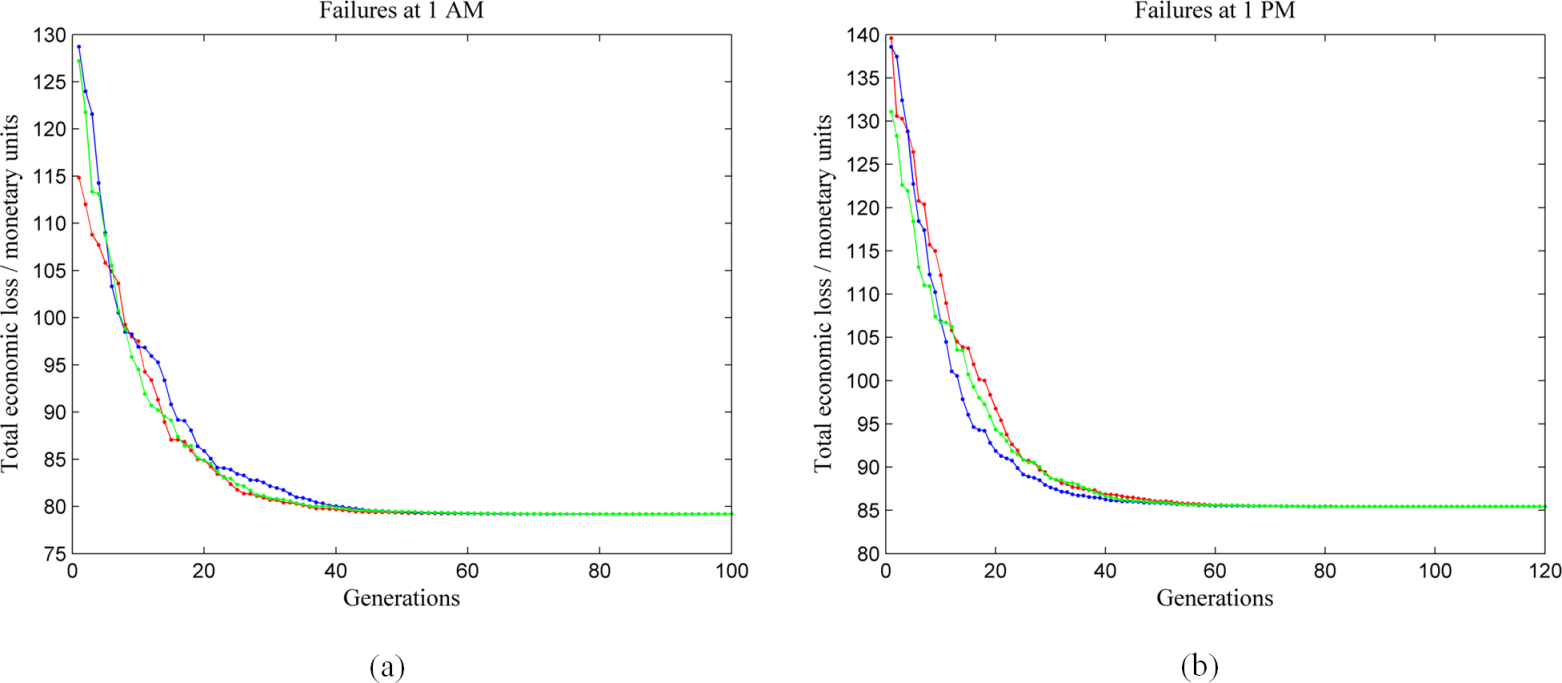
The fitness value during different generations. (a) failures happening at 1 AM; (b) failures happening at 1 PM.

To validate the joint restoration strategy model and examine the characteristics of optimal strategy, nodes {P19, P38, P54, P68, P85, P99, W19, W23, W41, W46} are supposed to encounter physical failure. For comparison, the failures are assumed to happen at 1 AM and 1 PM respectively. To solve the optimal restoration strategy with GA, we set the number of genotypes of individuals in initial generation as 40, 20 of them are chosen from feasible solutions, the others are randomly generated. The maximum generation is set as a number which makes the best impact area in each generation converge and not fluctuate for more than 30 steps. The crossover probability and mutation probability are set as 0.5 and 0.2. In Fig 7(A) and 7(B), the change of fitness value is shown for three separate calculations of failures happening at 1 AM (Fig 7(A)) and 1 PM (Fig 7(B)). The horizontal axis shows the number of iterations and the vertical axis shows the total economic loss (fitness value). It can be seen that, the change of fitness value decreases monotonically, and the total economic losses for three calculations converges to the same value after 60 generations.

The optimal restoration sequence solved for the failures at 1 AM and 1 PM are listed in Tables 3 and 4.

**Table 3:** Optimal restoration sequence for failures at 1 AM.

Time	Restored nodes	Economic loss(monetary units)	No. of failed nodes in electric power network	No. of failed nodes in water supply network
1AM		16.067	10	4
2 AM	P38, W19	17.317	9	7
3 AM	P19, P68	17.520	8	6
4 AM	P99, W23,W41	14.167	6	5
5 AM	W46, P85	8.293	4	4
6 AM	P54	5.575	2	3
7 AM		0.253	0	2
8 AM		0	0	0

**Table 4:** Optimal restoration sequence for failures at 1 PM.

Time	Restored nodes	Economic loss(monetary units)	No. of failed nodes in electric power network	No. of failed nodes in water supply network
1 PM		23.887	10	4
2 PM	P19, P38, W19	27.320	8	6
3 PM	P68, P99, W23,W46	21.392	7	5
4 PM	P54, P85, W41	12.606	3	3
5 PM		0.240	0	s2
6 PM		0	0	0

In Tables 3 and 4, the optimal restoration strategies are different when the same failures occur at different time. Comparing the two strategies, the strategy for failures at 1 PM is to complete the restoration in a shorter time. In Fig 4, the hourly power and water consumption is lower in the night and higher in the day. The consumer loss is lower if the failures occur at 1 AM. To reduce the restoration cost, the restoration strategy for 1 AM is to restore a small number of physically failed nodes at each time step, and restore all nodes in five time steps. In contrast, if the failures occur at 1 PM, a faster restoration strategy is selected. The optimal restoration strategy is to respectively restore 3, 4 and 3 physically failed nodes in following three time steps. The reason is that, consumer loss occupies a bigger portion in total economic loss when the failures happen in the daytime.

In Tables 3 and 4, no matter which strategy is selected, the numbers of failed nodes in two networks both first increase, and then decrease. The cause is that the physically failed nodes cause more nodes to functionally fail, so at the beginning, the numbers of failed nodes increase. After the beginning of restoration activities, numbers of physically failed nodes and functionally failed nodes gradually decrease to 0. In addition, for two strategies, there is a time delay for the recoveries of functionally failed nodes after restoration activities complete, because time is needed for service delivery to functionally failed nodes.

**Fig 8:**
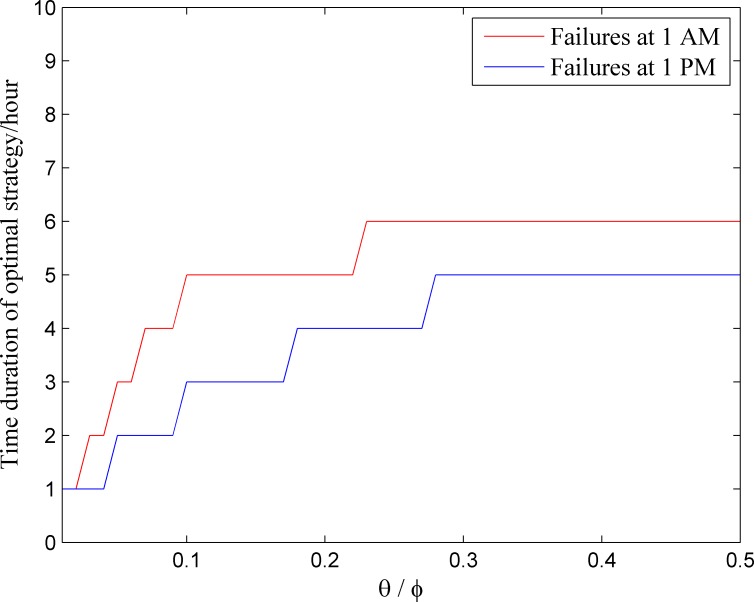
Time duration of the optimal strategy with change of *θ*/*φ*.

Sensitivity analysis of model parameters provides insight into how they affect model result. One basic finding from the case study is, if the failures occur at time with higher power and water consumption, quicker restoration should be conducted. The values of *θ* and *φ* reflect the basic properties of restoration cost and consumer loss for an infrastructure system. In this study, the sensitivity analysis is performed on the ratio *θ*/*φ* to the optimal restoration strategy. In following analysis, the duration of a strategy is defined as the time required for restoring physically failed nodes, e.g. the duration of optimal restoration strategy for failures at 1 AM is 5 time steps (see Table 3, from 2 AM to 6 AM). The assumptions that *θ*_*p*_ = 2*θ*_*w*_ and *φ*_*p*_ = 2*φ*_*w*_ are still valid. Fig 8 shows the duration of optimal strategy for failures happening at 1 AM and 1 PM with *θ*/*φ* (the values *θ*_*p*_/*φ*_*p*_ and *θ*_*w*_/*φ*_*w*_ are equal) ranging from 0.01 to 0.5.

In Fig 8, with same *θ*/*φ*, the duration of optimal joint restoration for failures at 1 PM is shorter than that for failures at 1 AM, consistent with former results. The duration of optimal recovery for failures occurring at time with higher power and water consumption is shorter. In Fig 8, no matter when the failures occur, if *θ*/*φ* is very small (less than 0.02), restoration of all physically failed nodes in one time step is the only option. Because the restoration cost is relatively small in comparison to the consumer loss, faster restoration could minimize the total economic loss from the infrastructure failures. With the increase of *θ*/*φ*, the duration of optimal recovery increases too. The cause is that, with the increase of *θ*/*φ*, the restoration costs will increase, and the consumer loss will decrease. If the increase of the restoration cost is larger than the decrease in consumer loss, slower restoration strategy will be selected. In addition, the change in the duration of optimal recovery strategy is marginally decreasing with respect to the increase of *θ*/*φ*. Therefore, although the time duration of optimal strategy increases with *θ*/*φ*, it is less sensitive for larger *θ*/*φ*.

## Conclusions

Services of infrastructure systems are essential to support the social and economic functioning of the society. In this paper, considering operating rules and interdependencies of infrastructure systems, a model for analyses of infrastructure interaction process is first proposed. It is argued that the model can capture the impacts of infrastructure failures in a predictive manner. Aiming to minimize the total economic loss from the infrastructure failures over time, a joint restoration strategy model is presented, which can determine the optimal joint restoration sequence at infrastructure components level. Through case study, we have demonstrated the validity of the model, and identified important factors for determining the optimal joint restoration strategy, such as failure occurrence time, etc.

There are also some limitations in the study. Above recommendations are largely dependent on modelling assumptions, such as the time step for failure propagation, functional dependencies between systems, etc. Although we believe these assumptions to some extent illustrate the real systems, their validity still need to be explored due to the complexity of real infrastructure systems. This study aims to present an analysis framework to address interdependent infrastructure restoration problem. If some assumptions are replaced in other forms, the proposed analysis framework still can help to determine the effective restoration strategy. Due to unavailability of detailed infrastructure data, the case study used in this research is illustrative. The future works will focus on the refinement of the approach for the application with real infrastructure systems.

## Supporting information

S1 File Program for generating infrastructure system topology. Please run the program in Matlab. Input the numbers of power nodes, power plant nodes, water nodes and water plant nodes.
(M) Click here for additional data file.

S2 File Data for Fig 4. Average power and water consumption of city Guilin in China.
(XLS)
